# The Training Characteristics of World-Class Distance Runners: An Integration of Scientific Literature and Results-Proven Practice

**DOI:** 10.1186/s40798-022-00438-7

**Published:** 2022-04-01

**Authors:** Thomas Haugen, Øyvind Sandbakk, Stephen Seiler, Espen Tønnessen

**Affiliations:** 1 School of Health Sciences, Kristiania University College, PB 1190, Sentrum, 0107 Oslo, Norway; 2 Department of Neuromedicine and Movement Science, Centre for Elite Sports Research, Norwegian University of Science and Technology, 7491 Trondheim, Norway; 3 School of Sport Sciences, UiT The Arctic University of Norway of Health Sciences, Tromsø, Norway; 4 Faculty of Health and Sport Sciences, University of Agder, PB 422, 4604 Kristiansand, Norway

**Keywords:** Endurance, Training periodization, Aerobic conditioning, Olympic athletes, Training logs

## Abstract

In this review we integrate the scientific literature and results-proven practice and outline a novel framework for understanding the training and development of elite long-distance performance. Herein, we describe how fundamental training characteristics and well-known training principles are applied. World-leading track runners (i.e., 5000 and 10,000 m) and marathon specialists participate in 9 ± 3 and 6 ± 2 (mean ± SD) annual competitions, respectively. The weekly running distance in the mid-preparation period is in the range 160–220 km for marathoners and 130–190 km for track runners. These differences are mainly explained by more running kilometers on each session for marathon runners. Both groups perform 11–14 sessions per week, and ≥ 80% of the total running volume is performed at low intensity throughout the training year. The training intensity distribution vary across mesocycles and differ between marathon and track runners, but common for both groups is that volume of race-pace running increases as the main competition approaches. The tapering process starts 7–10 days prior to the main competition. While the African runners live and train at high altitude (2000–2500 m above sea level) most of the year, most lowland athletes apply relatively long altitude camps during the preparation period. Overall, this review offers unique insights into the training characteristics of world-class distance runners by integrating scientific literature and results-proven practice, providing a point of departure for future studies related to the training and development in the Olympic long-distance events.

## Key Points

- This review bridges the gap between science and results-proven practice regarding how training principles and training methods should be applied for the Olympic long-distance events and identified clear distinctions in training organization between track runners and marathon specialists
- The weekly running distance is in the range 160–220 km for marathoners and 130–190 km for track runners, with both groups performing 11–14 sessions per week, and ≥ 80% of the total running volume at low intensity
- Training intensity distribution varies across mesocycles and differs between marathon and track runners, but common for both groups is that volume of race-pace running increases as the main competition approaches

## Background

Training for long-distance running (LDR) aims to improve the “big three” performance-determining variables: maximum oxygen uptake (VO_2_max; the highest rate at which the body can take up and utilize oxygen during severe exercise), fractional utilization (the ability to sustain a high percentage of VO_2_max when running), and running economy (VO_2_ at a given submaximal running velocity). Together, these variables integrate the sustained ability to produce adenosine triphosphate (ATP) aerobically and convert muscular work to power/speed [1–11]. International runners demonstrate different combinations of these determinants, as an “acceptable value” in one variable can be compensated for with extremely high values in the other variables. In addition, a “fourth variable,” neuromuscular power/anaerobic capacity, plays an important role in the decisive end phase of tactical track races [12]. Further, classic laboratory testing may not capture a “fifth variable,” fatigue resistance associated with specific adaptations that delay muscular deterioration and fatigue and enable maintaining race pace over the final 7–10 km of an elite marathon [13, 14]. Different time courses in the development of these performance determinants are very likely. This is exemplified by a case study of former marathon world record holder Paula Radcliffe who improved running economy by ~ 15% between 1991 and 2003, while $\dot{\text{V}}$O_2_max remained essentially stable at ~ 70 ml kg^−1^ min^−1^ [5].

Most world-class long-distance runners engage in systematic training for 8–10 years prior to reaching a high international standard [15]. Different pathways to excellence have been described, as both early and late specialization, and different backgrounds from other sports, can provide a platform for later elite LDR performance [15–18]. Several scientific publications during the last two decades have described the training characteristics of world-leading distance runners [17–31]. However, our understanding of best-practice LDR continues to evolve, and it is fair to say that positive developments in modern long-distance training methods have often been driven by experienced coaches and athletes rather than sports scientists [32]. Sport scientists have historically found themselves testing hypotheses regarding *why* elite athletes train as they do rather than driving innovation around the *how* in the training process. Tightly controlled and adequately powered laboratory studies that span the months-to-years timescales associated with maximizing all the above-mentioned physiological variables impacting LDR performance have been essentially infeasible if not impossible.

Publicly available coaching philosophies and training logs of podium contestants from international athletics championships and world marathon majors constitute a corpus of descriptive training information for the international long-distance community. It is tempting to call this corpus of information made available by international champions a description of training “best practice,” but some of our colleagues in the sports science community would reasonably argue that we can only know that this is *results-proven* practice, not if it is *best* practice. Combining and cross-checking data sources from available research evidence and results-proven practice provides a valid point of departure for outlining current training recommendations and for generating new hypotheses to be tested in future research [33–36]. This integrative approach also facilitates unique insights into training characteristics that previously have been scarcely investigated, altogether allowing a more holistic picture of “state-of-the-art” LDR training.

The objective of this review is therefore to integrate scientific and results-proven practice literature regarding the training and development of elite LDR performance. Within this context, we will particularly explore areas where the scientific literature offers limited information compared to results-proven training information. Moreover, the distinctions between training characteristics of the most successful marathon runners and track runners (i.e., 5000 and 10,000-m specialists) will be highlighted since they organize their training year differently. Although anchored in the standard Olympic running distances, this review is also relevant for other endurance sports.

## Methodological Considerations

The scientific literature supporting this narrative review was obtained from PubMed, using varying combinations of the search terms “endurance,” “long distance,” “marathon,” “training,” “conditioning,” “running,” “elite,” “high performing,” world-class,” “runners, ” and “athletes.” In addition, we searched for non-scientific, publicly available, and English-language training information related to podium contestants from international championships (i.e., Olympic Games [OG], World Championships [WC], and continental championships) and world marathon majors. Most of the training data were obtained from websites (Runner Universe, Sweat Elite, Running Science, LetsRun, and RunnersTribe) dedicated to providing the athletics community an expansive library of information written by top athletes and coaches. Within these websites, all relevant training logs and coaching philosophies were purchased/downloaded and reviewed. Training information from doping-banned athletes or coaches were excluded. Moreover, a Google Search for podium contestants (using athlete name and “training” as search terms) and LDR books was performed. Although we cannot guarantee that relevant data have not been overlooked, the search revealed training logs/information from 59 world-leading athletes and 16 coaches of podium contestants [15, 37–112] (Table 1). This information ranged from “typical training week” of various mesocycles to complete annual training logs. Interpretations of longitudinal training logs were weighted more heavily than “short-term” information. Similarly, training information from the 50 s, 60 s, and 70 s was mainly used to provide historical context.

**Table 1:** Sources of results-proven practice

Athletes [Ref.]	Personal bests (min)	International merits
Said Aouita ♂ [39]	5000 m 12:58.39 (WR)—mile 3:46.76	Olympic gold 1984, WC gold 1987
Stefano Baldini ♂ [69]	Marathon 2:07:22—Half marathon 1:00:50	Olympic gold 2004, EC gold 1998 and 2006
Dieter Baumann ♂ [40]	5000 m 12:54.70–3000 m 7:30.50	Olympic gold 1992, EC gold 1994
Kenenisa Bekele ♂ [81]	5000 m 12:37.35 (WR)—10,000 m 26:17.53 (WR)	3× Olympic gold and 5× WC gold 2003–2009
Joan Benoit ♀ [97]	Marathon 2:21:21—Half marathon 1:08:34	Olympic gold 1984
Gelindo Bordin ♂ [70]	Marathon 2:10:32—Half marathon 1:03:16	Olympic gold 1988, EC gold 1986 and 1990
Robert de Castella ♂ [82]	Marathon 2:07:51 (WR)	WC gold 1983
Joshua Cheptegei ♂ [41]	5000 m 12:35.36 (WR)—10,000 m 26:11.00 (WR)	Olympic gold and silver 2021, WC gold 2019
Stephen Cherono ♂ [60]	5000 m 12:48.81—3000 m SC 7:53.63 (WR)	WC gold 2003 and 2005
Constantina Diță ♀ [83]	Marathon 2:21:30—Half marathon 1:08:10	Olympic gold 2008, WC bronze 2005
Brendan Foster ♂ [62]	5000 m 13:14.6—10,000 m 27:30.3	Olympic bronze 1976, EC gold 1974
Haile Gebrselassie ♂ [42]	5000 m 12:39.36 (WR)—10,000 m 26:22.75 (WR)	2× Olympic gold and 4× WC gold 1995–2000
Sifan Hassan ♀ [49]	1500 m 3:51.95—10,000 m 29:36.67	2× Olympic gold 2021, 2× WC gold 2019
Takayuki Inubushi ♂ [71]	Marathon 2:06:57	Former Asian record holder in the marathon
Joyciline Jepkosgei ♀ [85]	Marathon 2:18:40—Half marathon 1:04:51 (WR)	WC silver 2018 and winner of New York marathon 2019
Steve Jones ♂ [80]	Marathon 2:07:13 (WR)	Winner of London and New York marathon in the 1980s
Deena Kastor ♀ [87]	Marathon 2:19:36—Half marathon 1:07:34	Olympic bronze 2004
Meb Keflezighi ♂ [78]	Marathon 2:09:08—10,000 m 27:13.98	Olympic silver 2004
Kip Keino ♂ [61]	5000 m 13:24.2—3000 m 7:39.6	2× Olympic gold and 2× Olympic silver 1968–1972
Bob Kennedy ♂ [43]	5000 m 12:58.21—3000 m 7:30.84	6th in the Olympics (1996) and WC (1997)
Sylvia Kibet ♀ [45]	5000 m 14:31.91—10,000 m 30:47.20	Olympic bronze 2008, WC silver 2009 and 2011
Eliud Kipchoge ♂ [76]	Marathon 2:01:39 (WR)—5000 m 12:46.53	Olympic gold 2016 and 2021, WC gold 2003
Florence Kiplagat ♀ [46]	Half marathon 1:05:09—10,000 m 30:11.53	WC gold 2009 and 2010 (cross-country and half marathon)
Wilson Kipsang ♂ [96]	Marathon 2:03:13—Half marathon 58:59	Olympic bronze 2012, 5 World Marathon Major wins
Abel Kirui ♂ [75]	Marathon 2:05:04—Half marathon 1:00:11	WC gold 2009 and 2011, Olympic silver 2012
Daniel Komen ♂ [57]	5000 m 12:39.74 (WR)—3000 m 7:20.67 (WR)	WC gold 1997
Brigid Kosgei ♀ [92]	Marathon 2:14:04 (WR)—Half marathon 1:04:49	Olympic silver 2021, 1st in four Marathon majors 2018–2020
Paul M. Kosgei ♀ [93]	Half marathon 59:07—10,000 m 27:21.56	WC gold (half marathon) 2002
Ingrid Kristiansen ♀ [63]	10,000 m 30:13.74 (WR)—Marathon 2:21:06 (WR)	WC gold 1987, EC gold 1986
Bernard Lagat ♂ [52]	5000 m 12:53.60—1500 m 3:26.34	2× WC gold 2007, Olympic silver 2004 and bronze 2000
Thomas Longosiwa ♂ [58]	5000 m 12:49.04—3000 m 7:30.09	Olympic bronze 2012
Tegla Loroupe ♀ [86]	Marathon 2:20:43—10 000 m 30:32.03	3× WC gold (half marathon) and 2× WC silver 1995–1999
Lisa Martin ♀ [88]	Marathon 2:23:51—10,000 m 31:11.72	Olympic silver 1988
Greg Meyer ♂ [79]	Marathon 2:09:01—10,000 m 27:53.1	Winner of Boston marathon 1981 and 1983
Geoffrey Mutai ♂ [73]	Marathon 2:04:15—Half marathon 58:58	Winner of New York, Boston and Berlin marathon 2011–2013
Imane Merga ♂ [59]	10 000 m 26:48.35—5000 m 12:53.58	WC bronze 2011, WC gold cross-country 2011
Lorraine Moller ♀ [97]	Marathon 2:28:17	Olympic bronze 1992
David Moorcroft ♂ [51]	5000 m 13:00.41 (WR)—3000 m 7:32.79	EC bronce 1978 and 1982
Moses Mosop ♂ [72]	Marathon 2:05:03—10,000 m 26:49.55	WC bronze 2005
Craig Mottram ♂ [53]	5000 m 12:55.76—3000 m 7:32.19	WC bronze 2005
Caleb Ndiku ♂ [55]	5000 m 12:59.17—3000 m 7:30.99	WC silver 2015
Yobes Ondieki ♂ [56]	10,000 m 26:58.38 (WR)—5000 m 13:01.82	WC gold 1991
Sonia O'Sullivan ♀ [48]	5000 m 14:41.02—3000 m 8:21.64	WC gold 1995, 3 × EC gold 1994–1998, Olympic silver 2000
Jim Peters ♂ [64]	Marathon 2:17:40	Four marathon WRs in the 1950s
Gordon Pirie ♂ [65]	5000 m 13:36.8—3000 m 7:52.8	Olympic silver 1956, EC bronze 1958
Paula Radcliffe ♀ [89]	Marathon 2:15:25 (WR)—10,000 m 30:01.09	WC gold, 3× WC half marathon gold, EC gold 2000–2005
Bill Rodgers ♂ [90]	Marathon 2:09:27 (WR)—10,000 m 28:04.42	Multiple winner of Boston and New York marathon 1976–1980
Rodgers Rop ♂ [94]	Marathon 2:07:32—Half marathon 1:00:56	Winner of New York and Boston marathon 2002
Molly Seidel [84]	Marathon 2:25:13—Half marathon 1:08:29	Olympic bronze 2021
Toshihiko Seko ♂ [91]	Marathon 2:08:27—10,000 m 27:42.17	Winner of Boston, London and Chicago marathon in the 1980s
Mubarak H. Shami ♂ [77]	Marathon 2:07:19—Half marathon 1:00:47	WC silver 2007, WC half marathon silver 2005
Charlie Spedding ♂ [74]	Marathon 2:08:33—10,000 m 28:08.12	Olympic bronze 1984
Ian Stewart ♂ [66]	10,000 m 27:43.03—5000 m 13:22.8	EC gold 1969, Olympic bronze 1972
Paul Tergat ♂ [54]	10,000 m 26:27.85—Marathon 2:04:55 (WR)	5× WC gold cross-country and 2× Olympic silver 1995–1900
Andy Vernon [50]	5000 m 13:11.50—10,000 m 27:42.62	EC silver and bronze 2014
Lasse Viren ♂ [67]	5000 m 13:16.4 (WR)—10,000 m 27:38.35 (WR)	4× Olympic gold 1972–1976, WC bronze 1974
Grethe Waitz ♀ [68]	Marathon 2:24:54 (WR)—Half marathon 1:07:50	WC gold 1983 and 5× WC cross-country gold 1978–1983
Susanne Wigene ♀ [47]	10,000 m 30:32.36—5000 m 14:48.53	EC silver 2006
Emil Zatopek [97]	5000 m 13:57.0—10,000 m 28:54.2	4× Olympic golds and 4× EC golds 1948–1954

Coaches [ref.]	Successful long-distance athletes	Athlete merits
Nic Bideau [20]	Craig Mottram	WC bronze 2005
Bill Bowerman [21]	Steve Prefontaine, Bill Dellinger, Matt Centrowitz	Bowerman trained 31 Olympic athletes
Antonio Cabral [22]	Alberto Chaica, Fernando Couto	Olympic and WC finals
Renato Canova [23, 24]	Abel Kirui, Sylvia Kibet, Imane Merga	45 Olympic/WC medals, 15 World Marathon Major wins
Jack Daniels [25]	Coached seven athletes to the U.S. Olympic team	Olympic finals
John Davis [26]	Dick Quax, Lorraine Moller	Olympic medals
Brad Hudson [27]	Dathan Ritzenhein	Olympic finals
Mihaly Igloi [28]	Multiple long-distance athletes in the 1950s and 1960s	A total of 49 world records
Arthur Lydiard [29–31]	Murray Halberg, Barry Magee	Olympic medals in the 1960s
Mihaly Iglói [28]	Sándor Iharos, Jim Beatty, Bob Schul	His athletes achieved 49 WRs in the 1950s and 1960s
Steve Magness [32]	Assistant coach and advisor for elite runners	Seven top-15 finishes at WC
Kim McDonald [33]	Daniel Komen, Stephen Cherono	Olympic and WC medals
Terrence Mahon [34]	Deena Kastor, Jen Rhines, and Ryan Hall	Olympic medals and finals
Gabriele Rosa [35]	Moses Tanui, Paul Tergat, Sammy Wanjiru	Olympic medals
Joe Vigil [36, 37]	Coach for the US Olympic team in 1998	Olympic finals
Chris Wardlaw [38]	Steve Moneghetti, Rob De Castella, Craig Mottram	WC medals

Overall, the 59 listed athletes have won 51 medals in Olympic Games (22 gold, 15 silver, 11 bronze), 62 medals in World Athletics Championships (26-14-17), 56 medals in continental championships (25-11-17), 25 medals in World Athletics Half Marathon Championships (15-3-1), 52 medals in World Athletics Cross Country Championships (31-8-9), 16 medals in World Athletics Indoor Championships (10-4-2) and 48 world marathon major wins. Eighteen of the listed athletes are former or current world record holders

*WC* world championships, *EC* European championships, *WR* former or current world record holder

Several limitations to our approach must be acknowledged. Firstly, the inclusion of results-proven training information can be discussed since it is not based on peer-reviewed research. However, elite athletes are systematic in their collection of training “data” and report their training accurately [23, 113], justifying the extensive use of training logs as primary or secondary information sources in scientific training characteristics studies within LDR [e.g., 17–28]. Secondly, an initial review of both the scientific literature and results-proven practice reveals several biases, including a substantial male dominance and focus on a few successful training groups. Additionally, the lack of a common framework (e.g., intensity zones) and terminology can result in misinterpretations. Moreover, the included literature cannot be controlled for possible training prescription–execution differences or changes in training programs over the years. We are also aware that many unsuccessful athletes have applied the same “recipe” as successful runners. Hence, we particularly focus on common key features across varying athlete groups. Finally, the widespread use of doping in international athletics must also be acknowledged [114, 115]. The outcomes of this review must therefore be interpreted with these caveats in mind. Sensitive to these limitations, we still contend that integrating scientific evidence and results-proven practice is a strong point of departure for outlining state-of-the-art training recommendations and for generation of new hypotheses to be tested in future research.

## Training Periodization and Competition Scheduling

Information about the periodization pattern of LDR training over the course of a year is scarce in the scientific literature. Since Arthur Lydiard introduced his periodization system in the late 1950s [46–48], leading practitioners typically divide the training year (macrocycle) into distinct, ordered phases (meso- or micro-cycles) with the explicit goal of peaking for major competitions [15, 21, 26–28, 39–57, 63, 67, 73, 76, 92, 94, 99, 100]. Because track and marathon specialists organize their training year and competition schedule quite differently, we will treat these groups separately in the remainder of this section.

At least three phases are typically organized within a macrocycle for track runners: a preparation period, a competition period, and a transition period. The transition period begins immediately after the conclusion of the outdoor competition season, typically consisting of 1–2 weeks with rest or recreational training/low-intensive running [15, 39–44, 49, 53–55, 63, 75, 87, 94], although some athletes may take ~ 4 weeks completely off [73]. The preparation period is typically broken up into *general* and *specific* preparation. In the general preparation period, the focus is high volume to build an aerobic foundation. From the specific preparation period onward, the focus gradually shifts toward higher volume of specific race-pace intensity [40–44, 49–56, 72, 73, 76, 92–94, 112]. Such organization of training has also recently been verified as highly effective in the research literature [116] and bears some resemblance with Matveyev’s traditional periodization model based on the training of successful Soviet athletes during the 1950s and 1960s [117]. While the Matveyev model suggested a dramatic shift from volume focus to intensity focus as the competition period neared, most track runners maintain a high volume of subthreshold endurance training throughout the preparation and competition period and are careful not to overuse race-pace training or introduce it too early in their annual cycle. This is somewhat in contrast to the research literature, where under-performance caused by overtraining/under-recovery tends to be closely associated with high volumes and/or densities of training rather than reduced volume and increased intensity [118].

Some track runners apply double periodization (i.e., two peaking phases), consisting of a preparation phase, an indoor or cross-country season, a new preparation phase, and finally an outdoor track competition season (typically lasting 3–4 months, starting in May and ending in September) [56, 57, 68]. However, most world-class track runners apply single periodization; they may participate in cross-country or indoor competitions during their preparation phase but use these competitions as part of their training. A review of the competition schedule for the athletes listed in Table 1 (based on their most successful year in an international championship) revealed that track runners participated in 9 ± 3 (mean ± SD) annual competitions, in which 6 ± 3 where outdoor races prior to OG or WC [119]. About half of the outdoor races were so-called “under-distances” (1500–3000 m), while the remaining half consisted of 5- or 10,000-m competitions. None of the analyzed track runners competed in “over-distances” (e.g., half-marathon) in the 3–4 preceding months leading up to the OG/WC. The last competition prior to OG/WC was performed 4 ± 2 weeks ahead, and 3 ± 2 additional competitions were performed in the subsequent 2–4 weeks after their most successful championship [119].

Marathon runners periodize their training year differently. The marathon runners listed in Table 1 participated in 6 ± 2 annual competitions in their most successful year, or ~ 50% fewer races than the track runners. These competitions were distributed across 2 ± 1 marathons (separated by at least 3 months), 1 ± 1 half-marathon(s), and 3 ± 3 races over 5–15 km [119]. Their last competition prior to OG/WC or a World Marathon Major was performed 10 ± 5 weeks ahead. Marathon runners typically apply double periodization centered around spring and autumn marathons, where the 7–14 days following the marathon competitions are completely training free or very easy [15, 112]. The 5–6 preceding months leading up to a marathon are typically divided into general and specific preparation [40–42, 52–54]. For track runners, the focus gradually shifts throughout the preparation period from achieving high total running volume to achieving more running volume at or near race pace. Progression is either based on extending the athlete’s accumulated session duration at a goal pace [40, 41] or establishing high intensity volume and then slowly increasing pace [92]. Some marathon runners even apply a reverse linear periodization model, with the highest running volumes registered during the preceding weeks of the tapering phase periods as the competition is approaching [112, 120].

The underlying mechanisms for the superiority of specific periodization models in LDR remain unclear, and there is no direct evidence enabling us to compare outcomes across various periodization methodologies [121]. Although scientific comparisons of different training approaches at a macro-level are challenging to perform, future studies should aim to verify and test the concepts developed by the best practitioners over the last decades.

## Training Methods

The specific training methods for LDR consist of varying forms of continuous long runs and interval training (Table 2). These training methods bear different labels among practitioners, mainly depending on the intention/goal of the training. For example, “easy runs” are somewhat misguidedly termed “recovery runs” or “regeneration” by some coaches [40, 41], assuming that their value is merely to “accelerate recovery” prior to the next hard session. No scientific studies to date support this assumption, but the feeling of recovery might be caused by the low load of such short easy runs, causing very little interference with the ongoing recovery process. However, accumulation of high frequency and volume of low-intensity training (LIT) is considered an important stimulus for inducing peripheral adaptations (e.g., increased mitochondrial biogenesis and capillary density of the skeletal muscle) [122]. Accumulated volume of low intensity running seems to be a characteristic of those with better running economy [123, 124], and continuous running is probably most beneficial in stimulating these adaptations [125]. High volumes of LIT likely promote better “neural entrainment,” decrease movement variability, and reduce energy cost of movement [126].

**Table 2:** Specific training methods for world-class long-distance runners

Training method	Description
*Continuous running*	
Warm-up/cooldown, easy run	Low-intensive running (typically 3–5 km h^−1^ slower than marathon pace, i.e., 3:45–4:30 and 4:15–5:00 min km^−1^ for men and women), however, the last part of the warm-up may approach marathon pace predominantly performed on soft surface (grass, woodland, forest paths, etc.). Typical duration for warm-up/cooldown is 10–30 min. Easy runs are typically applied prior to or after hard training sessions, typically lasting 40–70 min
Long run	Low-intensive steady-state running (~ 1–2 km h^−1^ slower than marathon pace, i.e., 3:05–3:30 and 3:30–4:00 min km^−1^ for men and women, with marathoners in the faster ends of these ranges). Typical duration is 45–120 min for track runners and 75–165 min for marathon runners. The running pace is not necessarily constant throughout the session. This training method is more specific for marathoners than track runners
Uphill run	Low-intensive steady-state running uphill (grades 3–6%). Typical duration 20–45 min (6–10 km)
Threshold run (also called tempo run)	A sustained run at moderate intensity/half-marathon pace (i.e., 2:50–3:05 and 3:05–3:30 min·km^−1^ for men and women). Typical duration 20–50 min (7–15 km). The session should not be extremely fatiguing
Fartlek	An unstructured run over varying terrain lasting 30–60 min, where periods of fast running are intermixed with periods of slower running. The pacing variations are determined by the athlete’s feelings and rhythms, and the terrain
Progressive long runs	A commonly used training form used by African runners. The first part of the session resembles an easy run. After about half the distance, the pace gradually quickens. In the final portion, the pace increases to half-marathon pace or slightly past it. Typical duration is 45–90 min. Athletes are advised to slow down when the pace becomes too strenuous
*Interval training*	
Threshold intervals (also called tempo intervals)	Intervals of 3–15 min. duration at an intensity around half-marathon pace or slightly faster. Typical sessions: 10–12 × 1000 m with 1 min. recovery or easy jog between intervals, 6–8 × 1500–2000 m with 1–2 min. recovery or easy jog between intervals, or 4 × 5000 m with 1000 m easy jog in between. Recommended total time for elite runners is 30–75 min. Such intervals are advantageous because they allow the athlete to accumulate more total time than during a continuous threshold run
VO_2_max intervals	Intervals of 2–4 min. duration at 3–10 K pace, with 2–3 min. recovery periods between intervals. Typical sessions: 4–7 × 800–1000 m or 2 × (6 × 400 m) with 30–60 s and 2–3 min. recovery between intervals and sets, respectively. Recommended total time for elite runners is ~ 15–20 min. This training method is more specific for track runners than marathoners
Lactate tolerance training	5000-m runners perform 1–2 weekly training sessions with high levels of lactate in the pre-competition and competition period. Such intervals typically range from 150 to 600 m at 800–1500 m race pace and 1–3 min. recoveries. Typical sessions: 10–16 × 200 m with 1 min. recovery between intervals, or 1–2 × (10 × 400 m) with 60–90 s and 3–5 min. recoveries between intervals and sets, respectively. Total accumulated distance ranges from 1500 to 8000 m in elite athletes
Hill repeats	The main intention is overloading horizontal propulsive muscle groups while reducing ballistic loading. Typical incline is 5–10%, and repetition duration vary from ~ 30 s to ~ 4 min. depending on intensity, goal (aerobic intervals, lactate production or tolerance training) and time of season. Typical sessions: 8–10 × 200 m with easy jog back recoveries, or 6–8 × 800–1000 m with easy jog back recoveries
*Speed work*	
Sprints	5–15 s runs with near-maximal to maximal effort and full recoveries. These can also be performed as strides, progressive runs, hill sprints or flying sprints, the latter where the rate of acceleration is reduced to allow more total distance at higher velocities. The main aim of the session is to develop or maintain maximal sprinting speed without producing high levels of lactate

The outlined running velocities across the varying methods are based on running at sea level in flat terrain. The exemplified sessions evolve throughout the training year, either in the form of duration, number of repetitions, running velocity and/or recovery time between repetitions (depending on the goal of the session)

Varying definitions of the term “threshold” are used in previously published literature. In this review, we refer to “threshold” as an intensity close to half-marathon pace. For elite runners, half marathon pace is at the upper end of the intensity range demarcated by LT_1_ and LT_2_ and approximates maximal lactate/metabolic steady state. This appears consistent with how distance runners interpret the term in practice

The historical view is that, compared to a high frequency of LIT bouts, high-intensity training (HIT) stimulates central adaptations to a larger degree (e.g., increased stroke volume of the heart) [127–129]. However, in well-trained athletes that are performing a high total volume of training, further increases in $\dot{\text{V}}$O_2_max are not consistently observed after periods of increased HIT [130–132]. However, there is growing evidence that HIT better stimulates peripheral adaptations in fast-twitch motor units via an adenosine monophosphate (AMP) sensitive signaling pathway [133, 134]. In sum, HIT and LIT seem to elicit a complex suite of overlapping and complementary adaptations [127, 135–137], justifying the judicious application of varying training intensities for performance development in LDR. Further, it is overly simplistic to dichotomize the LDR training process into “high volume” and “high intensity” phases or training bouts. Whether discussing LIT or HIT, resulting adaptive signaling and stress responses can only be understood when the context of accumulated duration is added. Bill Bowerman, co-founder of Nike and US coach at the 1972 Olympics in Munich where Frank Shorter won the marathon, summarized his training philosophy as follows: 2–3 weekly interval sessions, a weekly long run, and fill the rest with as much LIT as you can handle [15, 38]. This simple training description holds true for the training organization of most successful long-distance runners during the last 5 decades (see “Intensity distribution” section). However, while the interval sessions are considered “key” sessions for track runners, the training organization for marathoners is most often centered around their weekly “long runs.”

Several successful long-distance runners have supplemented their sport-specific training with alternative locomotion modalities, so-called cross-training, including swimming, biking, cross-country skiing, and workouts on elliptical machines [15, 39, 57, 94]. Arguments supporting the inclusion of cross-training include injury prevention and avoidance of training monotony [138, 139]. Because running is associated with lower total training duration and higher mechanical/ballistic load compared to other locomotion modalities [140], one could speculate if cross-training should be performed to a larger extent among highly trained long-distance runners to provide the same central and peripheral training stimulus with lower muscular mechanical load. Future long-term studies should aim to investigate the possible aerobic training effects of various types of cross-training.

Less specific training forms such as strength, power and plyometric training in small doses (relative to running training dosage) are commonly applied by world-leading long-distance runners [15, 44, 56–58, 60, 65, 70, 93, 94, 97, 104, 111]. Even though these training forms do not duplicate the holistic running movement, they likely target specific neuromuscular qualities that underlie running economy. A review of the results-proven practice shows that such supplementary training is typically implemented as a combination of (1) resistance training using free weights or apparatus (squats, cleans, lunges, step ups, leg press, etc.) without causing noteworthy hypertrophy, (2) circuit training with body mass resistance, (3) core strength/stability (e.g., sit-ups and back exercises), and (4) plyometrics in the form of vertical and/or horizontal multi-jumps on grass, inclines, stairs, hills (e.g., bounding, skipping, squat jumps) or jumping over hurdles [15, 44, 56–58, 60, 65, 70, 93, 94, 97, 104, 111]. Overall, this supplementary training is poorly described in terms of resistance loading, sets and repetitions, and caution must therefore be made when drawing conclusions. However, it appears that more strength, power and plyometric training are implemented during early-to-mid preparation (about twice a week) compared to the competition period (typically zero or one weekly session) [15, 44, 56–58, 60, 65, 70, 93, 94, 97, 104, 111]. Several studies have shown that strength, power and plyometric training 2–3 times per week can improve running economy in long-distance runners [11, 29, 141–143]. Paula Radcliffe improved her vertical jump performance from 29 to 38 cm between 1996 and 2003, a period where she improved her running economy and marathon performance considerably [5].

## Training Volume

Most world-leading marathon runners train 500–700 h year^−1^, while most corresponding track runners are in the range 450–600 h year^−1^ [15, 40–43, 54, 73, 76, 79, 87, 94]. The relatively broad ranges in training volume are also present in other endurance sports [132, 144–153] and are likely explained by individual differences in mechanical training load tolerance, intensity distribution, risk willingness, training age/career stage, application of cross-training, genetics and perhaps also psychological factors. The present training volume observations are in line with other studies of top-class long- and middle-distance athletes [19–21, 27, 28, 34], but a larger proportion of middle-distance training is devoted to strength, power, and plyometric training (particularly in 800-m runners) [34]. Successful endurance athletes in cross-country skiing, biathlon, cycling, triathlon, swimming, and rowing train considerably more (800–1200 h per year) [132, 144–153]. This is likely explained by the fact that LDR is a weight-bearing exercise where rapid plyometric muscle actions put high loads on muscles and tendons during each step. Accordingly, both total training volume and the duration of low-intensity sessions are relatively low for LDR compared to the other endurance sports [140]. To obtain a relatively high training volume, world-leading athletes seem to compensate by running twice a day most of the week [40, 41, 56–76, 79, 83–112].

Many long-distance runners accumulate much of their running kilometers on dirt roads/forest paths instead of paved roads to reduce mechanical loading and maximize training volume. This indicates that the running movement per se is not the main contributor to limited training tolerance, but rather the leg-surface interaction and resulting forces [140]. Running surface is a specific aspect of training periodization for marathoners. Because major marathons are performed exclusively on hard, paved roads, marathon specialists will build in continuous runs of increasing duration on asphalt or similar hard surfaces as they specifically prepare for these events [15, 41].

A discussion of training volume and the constraints created by mechanical interactions between runner and running surface would be incomplete without mentioning running shoes. Recent developments on the footwear front have received massive attention in the LDR community. The “super-shoe” was introduced to road running in 2016 and to track running in 2019, chronologically coincident with a wave of LDR records. These shoes are now subject to strict guidelines and testing [154]. The footwear features behind these performance improvements include shoe weight, material composition, heel thickness, and bending stiffness, altogether improving running economy (and thereby performance) significantly [155–158]. Importantly in the context of LDR training, anecdotal evidence (i.e., our discussions with national-level distance runners) also suggests less muscle soreness and increased training tolerance with the recent shoe technology, altogether facilitating slightly increased running volume. Future studies should investigate how the current rapid development in shoe technology will affect LDR training characteristics.

While most scientific studies tend to only report training volume across macro- and mesocycles [e.g., 17, 21, 27, 28], the results-proven practice describes more detailed fluctuations throughout the training year. Because most injuries are attributed to rapid and excessive increases in training load [159, 160], elite performers increase the total running volume gradually during the initial 8–12 weeks of the macrocycle. The initial training week is performed with ~ 40–60% of peak weekly running volume, increasing by ~ 5–15 km each week until maximal volume is reached [62, 63, 90, 94, 95, 100, 103]. This volume progression is mainly achieved by increasing training frequency in the initial phase, then subsequently raised further by lengthening individual training sessions. Variations in training volume progression rate seem to depend on training experience and individual predispositions. The younger the training age, and the longer the transition period, the more careful progression from early to mid-preparation within the macrocycle.

Typical weekly running volume in the mid-preparation period is ~ 160–220 km for marathon runners [15, 85–107, 111, 112] and 130–190 km for track runners [56–76, 112], distributed across 11–14 sessions. Peak weekly volume can reach 20–30 km higher values for both groups, but only for short periods (2–3 weeks) of time. These wide ranges must be interpreted in the context of running intensity. Some marathon runners cover “only” 130–150 km wk^−1^; however, a considerably higher proportion of their volume (25–30%) is at or near marathon race pace, compared to others who cover 220–240 km wk^−1^, with only 15–20% at or near marathon pace [85–107, 112]. Training volume in elite LDR increases ≤ 8–10% annually in their late teens and early 20s, before slightly declining and stabilizing in their mid-20s [17, 18, 49, 53, 54]. The difference in volume between marathon and track runners is mainly explained by fewer running kilometers per session for track runners, as training frequency is equal for both groups. As shown in Table 2, some long-run sessions for marathon runners may last up to 60 min longer compared to track runners.

One could argue that the ~ 10% slower running velocity in women [161] should be compensated for with less covered distance to ensure the same running duration between sexes. A counterargument is that men and women should apply equal distances during practice because they compete in the same disciplines [40, 41]. We observed no sex differences in distance covered among the track runners in this study. The analyzed female marathon runners covered ~ 5% (~ 10 km) less distance but trained 30–40 min wk^−1^ longer than males [85–107]. We can only speculate if the longer training duration is to compensate for the less covered distance.

Overall, total running volume has remained relatively constant among world-leading long-distance runners since the 1950–1960s [15, 46–48, 78, 80–82]. Some athletes have applied considerably higher volumes (≥ 300 km wk^−1^), seemingly experiencing more challenges related to injury management and fatigue [15]. Based upon both biomechanical and physiological factors, it is tempting to speculate that lighter athletes tolerate higher running volumes over time compared to their heavier counterparts. Assuming runners spend half the step cycle time on the ground, then the vertical forces exerted upon the ground must be twice the athlete’s body weight. Hence, the higher the body weight, the higher the impact forces during the landing phase. Moreover, slim runners possess superior thermodynamical conditions, as their sweat surface area to heat producing volume ratio increases with decreasing body size [162].

## Intensity Zones

While training volume in endurance sports is straightforward to quantify, training intensity quantification is more complicated. The preponderance of scientific and results-proven practice recommends that intensity scales/zones/domains in LDR should be based on physiological parameters (e.g., heart rate ranges, ventilatory/lactate thresholds), external work rates (running pace or types of training), or perceived exertion [17, 18, 21, 22, 25, 27, 28, 30, 40–42, 54, 112, 135, 163–165], but no consensus has so far been established. We would argue that this lack of consensus is consistent with an uncomfortable truth; no single intensity parameter performs satisfactorily in isolation as an intensity guide due to (1) intensity–duration interactions and uncoupling of internal and external workload, (2) individual and day-to-day variation, and (3) strain responses that can carry over from preceding workouts and transiently disrupt these relationships [13, 166, 167]. Consequently, combining external load, internal load, and perception regularly during training provides a triangulation of intensity characteristics that is probably complimentary and informative. Whatever intensity parameter that is chosen, describing and comparing training characteristics requires a common intensity scale. To address this, we have developed both a 3- and 7-zone intensity model (Table 3). These are mainly anchored around race pace and reflect the practices of world-leading track and marathon runners. In this way, we can analyze their training logs in more detail. Compared to our previously developed intensity scale for 800/1500-m specialists [34], this version was deemed more representative because (1) lactate production sessions are rarely performed in LDR, (2) long-distance runners present lower blood lactate values within each intensity zone, and (3) long-distance runners exhibit less pronounced velocity declines with increasing training/repetition duration. Admittedly, presenting two “customized” intensity scales when there is overlap among middle- and long-distance performers may be provocative, but we argue that the present scale better reflects the nature of long-distance training. Indeed, standardized intensity zone systems are imperfect tools and have been criticized for several reasons [34, 135, 168, 169]. However, the potential error sources seem to be outweighed by the improved communication between coach and athlete that a common scale facilitates [34, 135]. The intensity scale outlined here (Table 3) can be used as a framework for both scientist and practitioners involved in LDR.

**Table 3:** Intensity scale for long-distance runners

Scale		BLa	HR	VO_2_max	RPE	Pace reference	AWD	Int. time	Rec	Typical training methods
6-zone	3-zone	mmol·L^−1^	% max	%	6–20		min·session^−1^	min	min	
7	HIT	n/a	n/a	n/a	n/a	60–400 m	1–3	< 0:20	1–3	Maximal or progressive sprints, hill sprints
6	HIT	> 8.0	n/a	n/a	18–20	800–1500 m	5–20	0:30–2:00	0:30–3	Lactate tolerance training, hill repetitions
5	HIT	5.0–8.0	> 93	90–99	18–20	1500–5000 m	15–30	0:30–3	0:30–5	VO_2_max intervals, competitions, hill repetitions
4	HIT	3.5–5.0	88–92	85–89	16–18	10,000 m	20–35	3–6	1–5	VO_2_max intervals, hill repetitions, competitions
3	MIT	2.0–3.5	83–87	80–84	14–16	(Half) marathon^b^	30–60	6–20	1–3	Threshold runs/intervals, fartlek, competitions
2	LIT	1.0–2.0	73–82	70–79	12–14	n/a	20–150	n/a	n/a	Long runs, uphill runs, progressive runs^c^
1	LIT	< 1.0	60–72	55–69	9–12	n/a	20–150	n/a	n/a	Warm-up/cooldown^a^, easy long runs

BLa = typical blood lactate (normative blood lactate concentration values based on red-cell lysed blood); HR = typical heart rate; VO_2_max = maximal oxygen consumption; RPE = rating of perceived exertion; AWD = typical accumulated work duration; Int. = interval; Rec. = typical recovery time (active or passive) between repetitions; LIT = low-intensity training; MIT = moderate-intensity training; HIT = high-intensity training

^a^Warm-up is typically performed in zone 1–3, although with shorter duration, while cooldowns are typically performed in zone 1–2

^b^Progressive runs are typically performed in zone 1–3

^c^The difference between half-marathon and marathon speed is very small on an absolute scale among world-class long-distance runners. Hence, half-marathon pace represents the upper part of zone 3, while marathon pace represents the lower part of the same zone. It is also important to note that physiological measures (and RPE) normally “drift” upward considerably during a competition, reflecting a growing mismatch between internal and external load. For example, heart rate may increase ~ 20 beats per minute (and cross into “zone 4 or 5”) during the latter half of a marathon race. Hence, the stated values are meant as training guidelines. Finally, individual race pace evolves throughout the training year. For example, marathon pace may be 10–20 s slower per kilometer during early preparation period, meaning similar physiological stress when running at slower paces

Endurance athletes employ varying methods of intensity distribution quantification. These are anchored around blood lactate ranges, running pace references, “time-in-zone” heart rate analysis calibrated against preliminary threshold testing, or the “session goal” approach where each training session is nominally allocated to an intensity zone based on the intensity of the main workout part [112, 135, 164, 170]. The method of intensity quantification can affect the calculation of the intensity distribution [25, 168]. Based on the nature of available results-proven practice [15, 37–112], the time/distance-in-zone approach was applied in this review to assess the intensity distribution for the analyzed running sessions.

## Intensity Distribution

The description of training intensity distribution in previous studies of long-distance runners can mainly be categorized into the following three models: (1) The pyramidal model, characterized by a large volume of LIT combined with a small volume of moderate-intensity training (MIT) and an even smaller volume of HIT, (2) the polarized model, where the same large volume of LIT is combined with less MIT and more HIT, and (3) the threshold model, where a relatively larger proportion of training is performed in the threshold intensity range demarcated by lactate/ventilatory thresholds 1 (LT1/VT1) and 2 LT2/VT2 [17, 18, 21, 25, 26, 28, 112, 135, 163, 164, 170–172]. Indeed, these intensity distribution definitions have been argued to be vague and inadequate, forming a basis for misinterpretations [173, 174]. While previous studies have tended to focus on what model is most optimal for performance based on aggregated data for the entire training year [17, 18, 21, 25, 26, 28], the results-proven practice shows that athletes adjust intensity distribution modestly across meso- and micro-cycles (see later paragraphs in this section). It should also be noted that both MIT- and HIT-training sessions are psychologically and physiologically demanding, requiring increased recovery time between blocks or sessions compared to training at lower intensity. In this context, training at “moderate” intensity is relatively more metabolically demanding in highly trained endurance athletes because they can run at a very high percentage of their v$\dot{\text{V}}$O_2_max during MIT-sessions [6, 175].

The most consistent training intensity characteristic of elite distance runners is that most of the running distance (≥ 80%) is performed at low intensity throughout the training year (corresponding to zone 1 and 2 in our 7-zone scale) [15, 37–112], in line with previous research [15, 17–22, 25–28, 112, 135, 164, 168–172]. Most of this training is in turn executed in zone 1, and the duration of the easy runs is very stable throughout the training year. Because zone 2 is closer to marathon pace, a higher proportion of zone 2 is applied by marathon specialists, particularly during the specific preparation period [40, 41, 85–97, 100]. Weekly long runs are one of the most important sessions for marathon runners in this period [40, 41], typically performed as 30–40 km runs slightly below marathon pace. In contrast, an increasingly higher proportion of LIT is performed in zone 1 for track runners as the competition season approaches [41, 72–76].

Training in zone 3 (in the 6-zone scale) represents 5–15% of the total running volume in elite long-distance runners [15, 37–112]. However, this proportion can vary across meso- and micro-cycles. There is a trend among marathon runners toward performing a higher proportion of zone-3 training as the major competition approaches [40, 41, 85–97, 100]. Track runners seem to follow an opposite organization, as the highest amount of zone-3 training is performed in the early-to-mid preparation period, before decreasing when the competition season is nearing [41, 60, 72–76]. According to Casado et al. [17, 18], tempo runs (continuous running in zone 2–3 in our model) account for ~ 20% of the total annual running volume in world-class Kenyan long-distance runners, corresponding well with observations of Billat et al. [20] and data compiled here.

Interval training in zone 4–5 also represents 5–15% of the total running volume, but this proportion is inversely related to zone 3-training. That is, marathon runners perform most training in zones 4–5 in the early-to-mid preparation period before replacing such training with more extensive bouts of zone-3 and upper end of zone-2 training as the major competition approaches [40, 41, 85–97, 100]. In contrast, track runners increase the proportion of zone 4–5 training at the expense of zone 3 as the competition season approaches [41, 60, 72–76].

During the pre-competition and competition period, most world-class 5000-m runners perform 1–2 weekly interval training sessions in zone 6 or in combination with zone 5 [56, 68, 72–76]. These runners may perform 10–20 km weekly in zone 5–6 between May and August, while most marathoners avoid training with such high amounts of lactic/glycolytic energy release [40, 41, 85–97, 100].

Distance runners perform sprint training (zone 7 in our model) regularly during the annual cycle, although this accounts for less than 1% of the total running volume [15, 37, 40, 42–44, 49, 51, 54–60, 66, 68–76, 85, 88, 90, 91, 93, 94, 97, 102, 103, 105, 109–111]. Sprint training is considered a supplement rather than the main goal of separate training sessions and is typically performed during the last part of the warm-up or after easy long runs. It is generally assumed that sprint training should be performed without accumulation of fatigue (often indicated by increasing levels of blood lactate). The distances are most commonly in the range 60–120 m, with sufficient recovery between each repetition. Most sprint runs are performed with low to moderate rate of acceleration (i.e., strides, progressive runs, hills sprints, or flying sprints), likely because the energy demands during maximal acceleration greatly exceed those at peak velocity [176]. However, high amounts of endurance training limit the development of muscular power [177, 178], and it is unrealistic to expect significant sprint performance development in elite long-distance runners. Hence, sprint training is mainly performed to minimize the negative impact of aerobic conditioning on maximal sprint speed.

In summary, the annual training intensity distribution is very similar for track runners and marathon specialists, as low intensity volume dominates. However, substantial differences may be present within each mesocycle. Both groups increase the volume of race-pace running as the main competition approaches. Table 4 contrasts case study examples of typical training weeks across the annual cycle for a track runner and marathon specialist.

**Table 4:** Case study examples of training weeks for a marathon specialist and a track runner

Day	Eliud Kipchoge (gold medalist in Rio de Janeiro 2016 and Tokyo 2021 Olympics)	
	*General preparation period*	*Specific preparation period*
Mon	M: 16–21 km, average pace 3:50–4:00 min·km^−1^ (zone 1)	M: 21 km, average pace 3:20 min·km^−1^ (zone 2)
	E: 8–12 km, average pace 4:30–5:00 min·km^−1^ (zone 1)	E: 10 km, average pace 4:00 min·km^−1^ (zone 1)
Tue	M: 10–15 min warm-up (~ 3 km) (zone 1). 12–15 km interval training on a dirt track (e.g., 15 × 1000 m at 2:50–2:55 min·km^−1^ (zone 4) with 90 s rest	M: 3 km warm-up in 5:00 min·km^−1^ (zone 1). 1200 m in 3:25 min (zone 3), 5 × 1 km in 2:55 min (zone 3) with 90 s rest, 3 × 300 m in 42–40 s (zone 5) with 60 s rest, 2 × 200 m in 27 s (zone 5) with 60 s rest. 3 km cooldown in 5:00 min·km^−1^ (zone 1)
	E: 8–10 km, average pace 4:30–5:00 min·km^−1^ (zone 1)	E: Rest
Wed	M: 16–21 km, average pace 3:50–4:00 min·km^−1^ (zone 1)	M: 18 km, average pace 3:55–4:00 min·km^−1^ (zone 1)
	E: 8–12 km, 4:30–5:00 min·km^−1^ (zone 1)	E: 11 km, average pace 4:00 min·km^−1^ (zone 1)
Thu	M: 30 or 40 km long run, average pace 3:00–3:25 min·km^−1^ (zone 2–3), depending on terrain	M: 40 km tempo run (tough and muddy course), average pace ~ 3:40 min·km^−1^ (zone 1)
	E: 8–12 km, average pace 4:30–5:00 min·km^−1^ (zone 1)	E: Rest
Fri	M: 16–21 km, average pace 3:50–4:00 min·km^−1^ (zone 1)	M: 18 km, average pace 3:50–3:55 min·km^−1^ (zone 1)
	E: 8–12 km, 4:30–5:00 min·km^−1^ (zone 1)	E: 10 km, average pace ~ 3:55 min·km^−1^ (zone 1)
Sat	M: 50–65 min fartlek (zone 1–3), either with long intervals (e.g., 4 × 10 min with 2 min rest) or short intervals (e.g., 25 × 1 min with 1 min rest)	M: 85 min fartlek including 10 min warm-up at 5:00 min·km^−1^ (zone 1), 30 × 1 min at pace 2:45 min·km^−1^ (zone 4) with 1 min easy jog (zone 1) in between, 15 min cooldown (zone 1)
	E: 8–12 km, 4:30–5:00 min·km^−1^ (zone 1)	E: Rest
Sun	M: 18–22 km, average pace 3:50–4:00 min·km^−1^ (zone 1)	M: 20 km, average pace ~ 3:50 min·km^−1^ (zone 1)
	E: Rest	E: Rest
	*Weekly total of 200–220 km (82–84% LIT, 9–10% MIT, 7–8% HIT)*	*Weekly total of* ~ *185 km (*~ *91% LIT,* ~ *3% MIT,* ~ *6% HIT)*

Day	Thomas Longosiwa (5000-m bronze medalist in London 2012 Olympics)	
	*General preparation period*	*Competition period*
Mon	M: 15 km, average pace 4:00 min·km^−1^ (zone 1)	M: 20 km, average pace 3:45–3:50 min·km^−1^ (zone 1)
	E: 11 km, average pace 4:30 min·km^−1^ (zone 1). 10 × 80 m sprint uphill (zone 6)	E: 4 km warm-up in 5:00 min·km^−1^ (zone 1). 8 × 300 m steep uphill (zone 5)
Tue	M: 21 km, average pace 3:30 min·km^−1^ (zone 1–2)	M: 4 km warm-up in 5:00 min·km^−1^ (zone 1). 19 km fartlek with 7 km average pace 2:52 min·km^−1^ (zone 3), 6 km with average pace 3:24 min·km^−1^ (zone 2), and 6 km with average pace 3:50 min·km^−1^ (zone 1)
	E: 11 km, average pace 4:30 min·km^−1^ (zone 1)	E: 10 km, average pace 5:00 min·km^−1^ (zone 1)
Wed	M: 4 km warm-up in 5:00 min·km^−1^ (zone 1). 5 × 1000 m in 2:52 min (zone 4), 6 × 600 m in 1:38 min (zone 5), 7 × 300 m in 46 s (zone 5), 3000 m in 9:00 min (zone 3)	M: 18 km, average pace 4:10 min·km^−1^ (zone 1)
	E: 8 km, average pace 5:00 min·km^−1^ (zone 1)	E: 10 km, average pace 4:40 min·km^−1^ (zone 1)
Thu	M: 17 km, average pace 4:05–4:10 min·km^−1^ (zone 1)	M: 4 km warm-up in 5:00 min·km^−1^ (zone 1). 5 × 2000 m with alternating speed every 400 m, where a total of 6 km was performed with average pace 2:35–2:45 min·km^−1^ (zone 5). The remaining 4 km was performed with average pace 3:05–3:10 min·km^−1^ (zone 3)
	E: 11 km, average pace 4:30 min·km^−1^ (zone 1)	E: 10 km, average pace 5:00 min·km^−1^ (zone 1)
Fri	M: 15 km, average pace 4:00 min·km^−1^ (zone 1)	M: 18 km, average pace 3:40–3:45 min·km^−1^ (zone 1)
	E: 15 km, average pace 4:00 min·km^−1^ (zone 1)	E: 10 km, average pace 4:40 min·km^−1^ (zone 1)
Sat	M: 4 km warm-up, average pace 5:00 min·km^−1^ (zone 1). 12 km, average pace 3:06 min·km^−1^ (zone 3)	M: 4 km warm-up in 5:00 min·km^−1^ (zone 1). 3 × (5 × 600 m) in 1:33–1:34 min (zone 5)
	E: 11 km, average pace 4:30 min·km^−1^ (zone 1)	E: 12 km, average pace 4:10 min·km^−1^ (zone 1)
Sun	M: 24 km, average pace 3:50 min·km^−1^ (zone 1)	M: Rest
	E: Rest	E: Rest
	*Weekly total of 193 km (86% LIT, 8% MIT, 6% HIT)*	*Weekly total of 163 km (85% LIT, 7% MIT, 8% HIT)*

Their training was performed in hilly terrain on uneven surface at 2000–2500 m altitude. The training data of Thomas Longosiwa were provided by his coach Renato Canova, while the training data of Eliud Kipchoge are publicly available [76]

*M* morning session, *E* evening session, *z* training zone (see this table)

## Tapering

Tapering in elite sports refers to the marked reduction of total training load prior to important competition(s). This is a short-term balancing act, as tapering strategies are intended to decrease the cumulative effects of fatigue while maintaining fitness [179, 180]. Because tapering strategies and outcomes are heavily dependent on the preceding training load, it is often challenging to separate tapering from periodization and training programming in general. According to previous research, a successful taper may enhance competition performance in well-trained endurance athletes by ~ 1–3% [179–182]. However, this claim is challenging to verify in elite LDR, as numerous confounding external variables (race tactics/pacing, weather conditions, competitors, etc.) influence performance in many important competitions where runners compete for medals and not for the best possible time [183–185]. It has also been shown that outstanding performances across a 3-month competition period can be achieved, without tapering for a specific competition, by merely reducing the training substantially in the last 4–5 days prior to each competition [73].

In cases where major competitions are arranged in warm and/or humid cities, and perhaps also many time zones away from the athletes’ regular location, tapering is integrated with time-, heat-, and humidity-acclimatization processes. For more details related to these topics, we refer readers to previously published reviews [186–188].

The general scientific guidelines for effective tapering in endurance sports include a 2- to 3-week period with 40–60% reduction in training volume adopting a progressive nonlinear format, while training intensity and frequency are maintained [179–182]. However, most long-distance runners do not report a substantial decrease in training volume until the last 7–10 days prior to competition [61, 69, 74, 75, 85–95, 97]. Table 5 presents training volume distribution across intensity zones for 10 world-class marathon runners during the countdown to a major competition.

**Table 5:** Training volume across intensity zones for 10 world-class marathon runners during the countdown to a major competition

	Week 5	Week 4	Week 3	Week 2	Week 1^a^
Total volume	191 ± 29	184 ± 24	188 ± 17	170 ± 30	116 ± 27
Zone 1	150 ± 29	138 ± 22	150 ± 22	134 ± 30	98 ± 22
Zone 2	18 ± 15	27 ± 15	11 ± 13	13 ± 13	5 ± 5
Zone 3	17 ± 8	12 ± 9	21 ± 11	16 ± 15	10 ± 12
Zone 4	3 ± 7	7 ± 7	5 ± 6	5 ± 4	2 ± 2
Zone 5	2 ± 4	1 ± 2	0 ± 1	2 ± 4	2 ± 2

All data are stated in km (mean ± SD)

^a^Major competition not included. Zone 6–7 training accounted for < 0.5 km on average in these weeks. The data are collected from training logs from the following athletes (and competitions): Stefano Baldini (Olympic gold in Athens 2004 with 2:10:55), Kenenisa Bekele (winner of Berlin Marathon 2019 with2:01:41), Gelindo Bordin (Olympic gold in Seoul 1988 with 2:10:32), Takayuki Inubushi (2nd in Berlin Marathon 1999 with 2:06:57), Meb Keflezighi (winner of Boston Marathon 2014 with 2:08:37), Eliud Kipchoge (winner of Berlin Marathon 2017 with 2:03:32), Abel Kirui (World Championship gold in Daegu 2011 with 2:07:37), Moses Mosop (2nd in Boston Marathon 2011 with 2:03:06), Geoffrey Mutai (winner of New York Marathon 2011 with 2:05:05), Mubarak Hassan Shami (winner of Paris Marathon 2007 with 2:07:17)

A review of the competition schedule for the athletes listed in Table 1 (based on their most successful year in an international championship) revealed that the last competition was performed 10 ± 5 and 4 ± 2 weeks prior to the season’s main competition for marathon runners and track runners, respectively [119]. Arrival at the championship destination typically occurs 7–10 days ahead of competition [39, 54, 57, 94]. The last intensive session (e.g., 10 × 200 m at race pace with optional recoveries) is typically performed 3–5 days ahead of the main championship event [40, 61, 74, 75, 100].

## Altitude Training

The LDR community became aware of the impact of altitude on endurance performance in the late 1960s and particularly in connection with the 1968 Olympics in Mexico City (2300 m above sea level). Clearly, sufficient altitude acclimatization ahead of endurance competitions at altitudes 1000 m above sea level is required to perform optimally [189, 190]. However, many athletes additionally use longer sojourns at altitude to enhance aerobic endurance capacity and thereby performance at sea level, mainly with the goal of increasing red blood cell mass [191]. Since 1968, > 90% of all OG/WC medals from the 800 m through the marathon have been won by athletes who have lived or systematically trained at altitude [9, 15, 103].

The potential effect of altitude training is influenced by the hypoxic dose, which is a function of the duration of the stay and the altitude [192]. Most world-class African runners apply the "live high—train high” model, as they live and carry out LIT-, MIT-, and HIT-sessions relatively high (2000–2500 m above sea level) [9]. Athletes from lowlands typically perform relatively long altitude camps during the preparation period and one camp 2–4 weeks prior to the most important competition, with most emphasis on LIT and MIT-sessions [57, 85, 100, 103, 111]. However, the optimal time of return from altitude camps to lowland competition is disputed [193] and warrants further investigations. The ability to train effectively at altitude may be one feature that distinguishes African runners from their European, American, and Asian competitors [9]. In all cases, successful use of altitude training by the best long-distance runners is characterized by individualized, well-balanced training load and optimized recovery strategies through adequate sleep, rest and nutritional factors as described in detail elsewhere [e.g., 19, 194].

It has been questioned whether altitude training has positive effects on endurance capacity and sea-level performance beyond the effects achieved with similar training performed at sea level. Here, high-quality scientific evidence is limited, and researchers interpret the current scientific data differently [195, 196]. Altitude training research is methodologically demanding due to the difficulty of standardizing the intervention, including control groups, and controlling other psychological and physiological confounders during altitude training. Although research provides limited support for a positive effect of altitude training on sea-level performance in endurance sports, these studies remain scarce and underpowered to detect the small adaptations that may be of importance in elite LDR. This is illustrated through the large individual differences in blood responses documented in connection with altitude training [197]. Consequently, a nuanced view on altitude training is warranted.

## Conclusions

This review integrates the scientific literature and results-proven practice regarding the training and development of world-class LDR performance. Herein, we have outlined a framework for specific characteristics (i.e., training methods, volume, and intensity) and identified the training organization differences between track runners and marathon specialists. Marathon and track runners participate in 6 ± 2 and 9 ± 3 (mean ± SD) annual competitions, respectively. Typical weekly running volume in the mid-preparation period is in the range 160–220 km for marathon runners and 130–190 km for track runners. These differences are mainly explained by fewer running kilometers for each session for track runners, as training frequency (11–14 sessions per week) is equal for both groups. Moreover, ≥ 80% of total running distance is performed at low intensity throughout the training year. In the general preparation period, the focus is to build an aerobic foundation by a large total running volume. From the specific preparation period onward, the volume of race-pace running increases as the main competition approaches. Hence, training intensity distribution models vary across mesocycles and differ between marathon and track runners. While the African runners live and train at high altitude (2000–2500 m above sea level), most lowland athletes apply relatively long altitude camps during the preparation period. The tapering process starts 7–10 days prior to the main competition, typically preceded by a 2–4-week altitude camp. Overall, this review offers novel insights into areas of LDR training that formerly have been scarcely studied in the scientific literature, providing a point of departure for future studies and may serve as a position statement related to the training and development in the Olympic long-distance events.

## Data Availability

All data and materials support the published claims and comply with field standards.
